# The Effect of Job Skills and Job Burnout on Job Satisfaction Among Health Information Management Staff

**DOI:** 10.1002/hsr2.70217

**Published:** 2024-12-04

**Authors:** Mahdie Shojaei Baghini, Dorsa SepehryRad, Fatemeh Miri, Fatemeh Asadi

**Affiliations:** ^1^ Medical Informatics Research Center, Institute for Futures Studies in Health Kerman University of Medical Sciences Kerman Iran; ^2^ Department of Health Information Technology, School of Paramedical Zahedan University of Medical Sciences Zahedan Iran; ^3^ Faculty of Management and Medical Information Sciences Kerman University of Medical Sciences Kerman Iran

**Keywords:** health information management, job satisfaction, job skills, professional burnout

## Abstract

**Background and Aims:**

The health information management department faces multiple challenges such as multiple tasks, high workload, role ambiguity and conflict, and work pressure. In addition, informatics and health information technology tools are continuously used in this sector. Based on studies, the use of information technology affects job burnout and job satisfaction. Since no similar study has been conducted in the health information management department, this study was conducted to investigate the relationship between job skills, job burnout, and job satisfaction in health information management departments.

**Methods:**

In this cross‐sectional study, the sample consisted of 201 personnel from the health information management department. A systematic random sampling method was used to select the participants. Data was gathered by using a questionnaire and visiting the selected hospitals in Kerman and Zahedan. For data analysis, SPSS 20 and SmartPLS 3 were used. PLS path models are fundamentally composed of the measurement and the structural model. To evaluate the measurement model, the formative measurement model tests were evaluated. Also, the hypotheses test, VIF, and CV‐Red tests were used to evaluate the structural model. Then, the evaluation of the quality and suitability of the model was evaluated with the model fit test.

**Results:**

The results showed that positive and significant job skills were associated with job satisfaction and a negative and significant relationship with job burnout. Also, job burnout does not play an intermediary in this context. In addition, the CV‐RED values for all three variables were the moderate range. The SRMR value of 0.120, which is in the medium to high range, indicates an adequate fit of the model.

**Conclusion:**

These findings provide useful information for decision‐makers and human resource managers in the health sector and suggest practical strategies for reducing job burnout and increasing job satisfaction among health information management staff.

## 1 Introduction

Employees are any organization's most important strategic resource and are key to its success [1]. Their perceptions and attitudes towards their jobs influence the quality and quantity of work outcomes. This phenomenon is known as “job satisfaction” [2]. Job satisfaction reflects employees' feelings about work tasks, conditions, rewards, and relationships [3]. It significantly impacts various individuals and organizational behavior and performance aspects [4]. From an organizational perspective, high job satisfaction indicates a positive organizational climate that attracts and retains talented employees. One of human resource management's primary responsibilities is maximizing employee job satisfaction [5, 6]. Therefore, identifying factors that contribute to or hinder job satisfaction is essential [7].

Previous research has demonstrated a relationship between job satisfaction and job skills [7, 8, 9, 10]. Job skills encompass the mental or motor skills required for specific job tasks [7]. According to social cognitive theory, organizational members need to believe in and master their job tasks to achieve desired job performance. As a result, high‐skilled employees are more resilient to stress and job burnout, less prone to misconduct, and more likely to maintain their creativity and organizational behaviors. In contrast, low‐skilled employees often engage in counterproductive behaviors due to their poorness in management and decision‐making [5, 6, 7, 8, 9, 10, 11].

The relationship between job satisfaction and job burnout is examined based on Frederick Herzberg's hygiene‐motivation theory [12]. Job burnout, a chronic stress syndrome characterized by chronic fatigue, a negative attitude towards work (cynicism), and reduced professional efficiency, has recently garnered special attention from psychologists and managers [13]. Job burnout is any mental or physical exhaustion resulting from job duties, is common among employees and workers in the modern labor market, and is not limited to any specific group [14, 15].

Although job burnout is related to job satisfaction, many factors influence this relationship, such as high workload, role ambiguity, role conflict, stressful events, and work pressure [16, 17, 18, 19]. In different studies, some of these factors have been investigated. For example, survey data from 355 hotel employees in Taiwan shows that job skills is positively and significantly associated with job satisfaction, while job burnout weakens the relationship between job skills and job satisfaction [20]. Additionally, perceived organizational support influences the magnitude of the effects of job skills, job burnout, and satisfaction on each other [20]. Similarly, the findings of Davali et al. revealed that job burnout indirectly mediates the relationship between job skills and job satisfaction [5]. Furthermore, information technology impacts job burnout because techno‐stressors significantly influence work exhaustion [21, 22]. The information technology worker's environment—knowledge‐oriented work, around‐the‐clock service, and requirements for creative solutions to business issues—has the potential to create work exhaustion, triggering depersonalization and reduced achievement [23, 24].

With the expansion of the computerization of health information, informatics tools and health information technology are constantly used in health information management departments [25]. Also, this department ncounters numerous challenges, including various tasks, high workload, role ambiguity, conflict, stressful events, and work pressure. Organizational support was found to be more critical for nonclinical employees compared to clinical ones. This is noteworthy because nonclinical employees, who also had to cope with increasing workloads and long hours, may have received less recognition compared to frontline staff [26]. The primary objective of this study is to investigate the structural model of the effect of job skills on job satisfaction and job burnout in the health information management departments of hospitals. The main hypotheses of the research are the effect of job skills and job burnout on job satisfaction with the mediating role of job burnout of health information management employees. To the best of the authors' knowledge, this is the first study done in hospitals' health information management department. As health information management increasingly becomes a pivotal aspect of global healthcare systems, this study contributes to a deeper understanding of how job skills and burnout interact to shape job satisfaction among these vital employees. The findings of this study can be used as empirical evidence to inform policymakers in enhancing the job satisfaction of the human resources of these hospitals.

We present this article in accordance with the STROBE reporting checklist.

## 2 Methods

### 2.1 Study Design

This cross‐sectional study was carried out in the health information management department of Kerman and Zahedan hospitals from June to August 2023. It employed a quantitative approach and focused on staff members working in these departments. The inclusion criteria were having at least 1 year's experience in any unit of the health information management department and consent to participate in the study.

### 2.2 Data Collection

**Figure 1:**
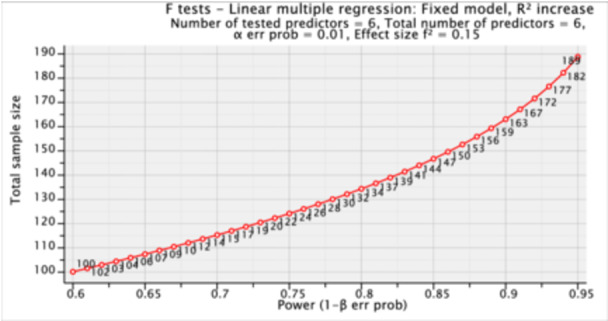
Sample size.

For data collection, the sample size was calculated using the G‐Power version 3.1 software, resulting in a total of 189 individuals. The parameters set for this calculation were an alpha level (α) of 0.01, a statistical power of 0.95, an effect size of 0.15, and six predictor variables. To compensate for potential non‐responses or dropouts, an additional 10% of questionnaires were distributed (as illustrated in Figure 1). A systematic random sampling technique was employed to select participants. Data gathering was conducted through visits to the hospitals as mentioned earlier.

### 2.3 Instrument Development

The scales utilized in this research were previously established. Considering that the questionnaires are designed generally, the core of the questions according to the research population aligned with the study's focus. Job burnout was evaluated using the Maslach Job Burnout Questionnaire, comprising three dimensions: emotional exhaustion (nine items), depersonalization (five items), and reduced personal accomplishment (eight items), was implemented [27]. The short form of the Minnesota Job Satisfaction Questionnaire (MSQ) was used to measure employee job satisfaction. The short form of MSQ through a six‐item scale of payment system (three items), job type (four items), advancement opportunities (three items), organizational climate (two items), leadership style (four items), and physical conditions (three items) measures the satisfaction of personnel [28]. The job skills assessment consisted of 13 items adapted from MafakheriBashmaq. It was categorized into searches for resources (six items), looking for challenges (three items), and reducing demand (four items) [29].

### 2.4 Data Analysis

For data analysis, SPSS 20 was utilized alongside structural equation modeling (SEM) to assess variable relationships and hypothesis testing. The hypotheses were evaluated with a P value of 0.05. Given the model's incorporation of a mediator variable, SmartPLS 3 was employed for confirmatory factor analysis and model validation, suitable for analyzing complex models including second‐order and bifactor structures [30]. PLS path models are fundamentally composed of two linear equation sets: the measurement (or outer) model and the structural (or inner) model [31]. The initial step in PLS analysis involves evaluating the measurement model. This model is instrumental in quantifying the associations between observed variables and latent constructs [32]. The Inner Model elucidates the interrelationships among the latent variables [33]. The structural model elucidates the significance of the relationships within the structure, and assuming the significance of these hypotheses, it helps determine the strength and direction of the effects exerted by the latent variables [34]. To evaluate the measurement model in this study, as all the variables are formative, only the formative measurement model tests were evaluated [32]. In formative measurement models, the multicollinearity of indicators is a critical issue, therefore, the variance inflation factor (VIF) test was applied [33]. Also, the hypotheses test, VIF and cross‐validation redundancy (CV‐Red) tests were used to evaluate the structural model. Ensuring that the independent variables in the study are not collinear is essential [35]. Ringle highlights that VIF values are the most reliable indicators for detecting the absence of collinearity among independent variables [36]. CV‐Red is a crucial metric in the context of PLS path modeling, especially during the process of cross‐validation. This metric assesses the degree to which a latent construct predicts other constructs in the model. A high CV‐Red value indicates that the latent variable in question possesses strong predictive capabilities regarding the endogenous variables in the model. On the other hand, a low CV‐Redundancy value points to a relatively minimal contribution of the latent variable in predicting the outcomes of other variables within the model. This measure is essential for ensuring the robustness and relevance of the latent constructs in PLS path modeling, making it a vital aspect for consideration and analysis in scientific research [37, 38]. After assessment of the measurement and structural model, it's essential to appraise its quality and suitability. Model fit is used to gauge how well a theoretical model aligns with an empirical one [39].

### 2.5 Ethical Approval

Ethical considerations were diligently followed. Verbal informed consent was secured from each participant before enrollment. Participation was voluntary, with a thorough explanation of the study's objectives and methods. Confidentiality of all data was ensured. The study received ethical approval from the Research Ethics Committee of Kerman University of Medical Sciences, Kerman, Iran (IR. KMU. REC.1402.026).

## 3 Results

### 3.1 Sample Characteristics

Among 201 health information management workers who participated in our study revealed that 63.7% were female. In terms of demographics, the largest age group was 20–30 years old, comprising 61.2% of the participants, and the same percentage held a bachelor's degree. Regarding professional experience, the predominant group, accounting for 41.8%, had less than 5 years of experience in health information management. Within this department, the hospital admission unit was the most common workplace, with 29.9% of participants employed there, as detailed in Table 1.

**Table 1:** Basic characteristic of participants.

Variables	Categories	Frequency	Percent
Gender	Female	128	63.7
	Male	73	36.3
Age	20–30	123	61.2
	30–40	60	29.9
	40–50	17	8.5
	> 50	1	0.5
Education	Associate degree	27	13.4
	Bachelor's degree	123	61.2
	Master's degree and PhD	51	25.4
Experience	< 5	84	41.8
	5–10	69	34.3
	10–20	32	15.9
	20–30	16	8.0
	< 5	84	41.8
Workplace	Head of department	14	7.0
	Admission	60	29.9
	Filing	36	17.9
	Coding	50	24.9
	Statistics	33	16.4
	Department secretary	8	4.0

The mean job skill of personnel was 3.4, job burnout was 2.6, and job satisfaction was 2.4. The descriptive statistics of the details related to each item of the variables are listed in Table 2.

**Table 2:** Descriptive information test results.

Variable	Item	Mean	Standard Deviations	Ranges
job satisfaction	payment system	2.1758	.76161	1‐5
	job type	2.7475	.74498	1‐5
	advancement opportunities	2.0050	.73218	1‐5
	organizational atmosphere	2.6567	.71698	1‐5
	leadership style	2.7326	.69305	1‐5
	physical conditions	2.4262	.87191	1‐5
job satisfaction (total)		.47049	2.4573	
job burnout	emotional exhaustion	3.0039	.68713	1‐5
	depersonalization	2.1055	.61808	1‐5
	personal accomplishment	2.8252	.53474	1‐5
job burnout (total)		.33855	2.6449	
job skills	searches for resources	3.3706	.61745	1‐5
	looking for challenges	3.7446	.69122	1‐5
	reducing demand	3.1704	.62640	1‐5
job skills (total)		3.4286	.44513	

### 3.2 Measurement Model Analysis (Outer Model)

According to Tenenhaus, VIF value below five is considered acceptable, while a value below two is deemed excellent [36]. VIF results showed that although all the formative constructs were acceptable, most of them were in the excellent range.

### 3.3 Structural Model Analysis (Inner Model)

**Figure 2:**
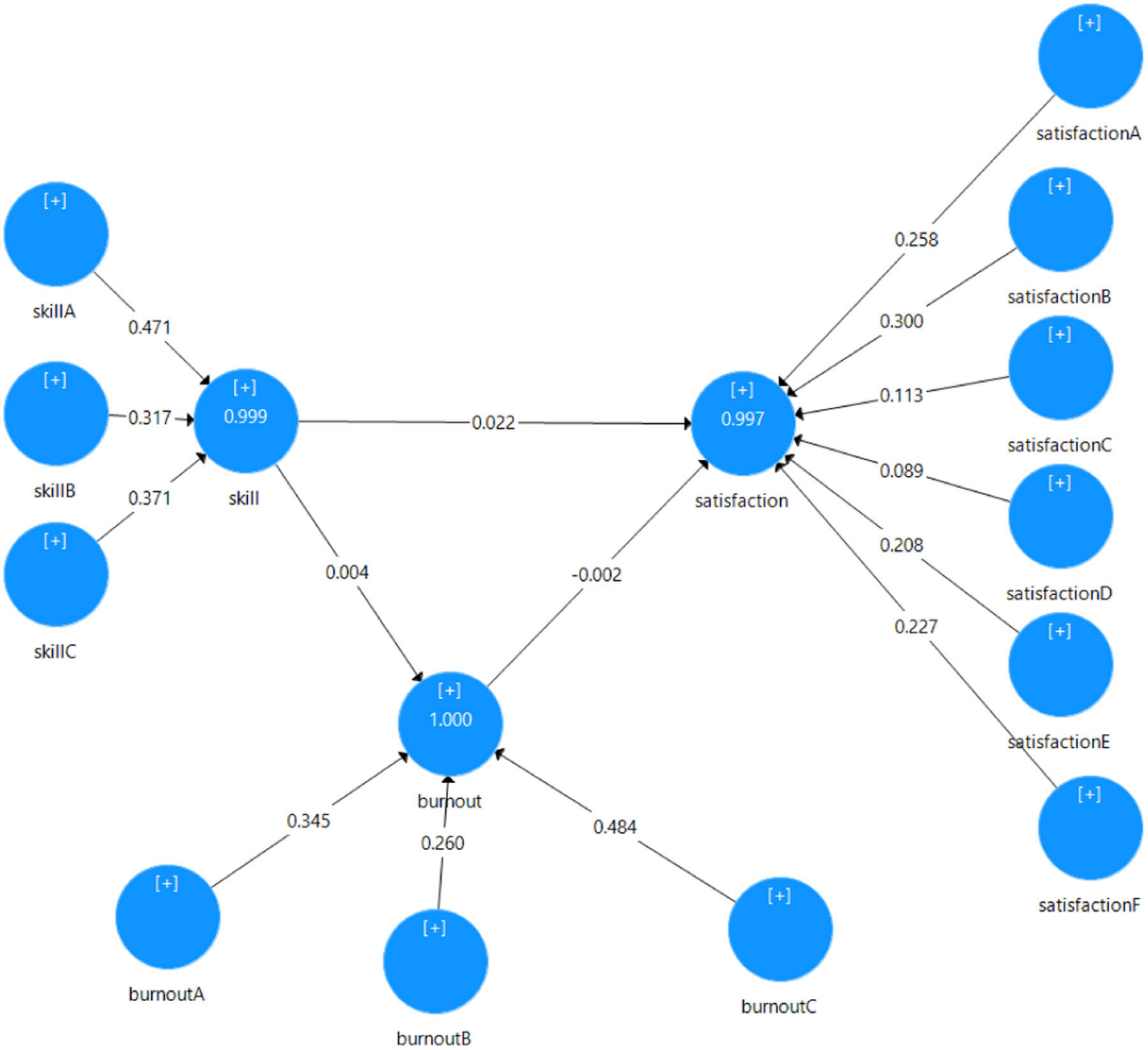
Structural model with significance coefficients.

Figure 2 illustrates the Structural model with significant coefficients. Considering that the variables were dimensional, the Figure specifies the dimensions of each variable. The instrument development section gives more complete explanations about the dimensions of the variables.

### 3.4 Hypotheses Tests

In this study, at the 95% confidence level, two of the three structural relationship hypotheses were significant due to bootstrapping. Job skills significantly affected job burnout (*p* = 0.026) and job satisfaction (*p* = 0.041). In this case, there was no significant relationship between job burnout and job satisfaction (*p* = 0.873). According to Chin, the intensity of the effects in two significant hypotheses is considered weak [34]. Both hypotheses had a positive relationship (Table 3).

**Table 3:** Hypotheses test results.

Hypothesis	Relationship	Path Coefficient	P‐value	T‐value	Status
H1	job skills ‐> job satisfaction	0.022	0.041	2.052	Supported
H2	job skills ‐> job burnout	0.004	0.026	2.537	Supported
H3	job burnout ‐> job satisfaction	−0.015	0.873	0.161	Not Supported

In this research, the collinearity within the structural model was rigorously evaluated. Echoing Tenenhaus (2005), a VIF below five is acceptable for questions, while a value under two is considered ideal [36]. The study found that the uncollinearity of Job skills with Job burnout was below two, thus meeting the criteria for being ideal. Also, uncollinearity of exogenous variables for job satisfaction was acceptable, as illustrated in Table 4.

**Table 4:** Collinearity of structural model of exogenous variables.

	Job satisfaction	Job burnout
Job skill	3.86	1.75
Job burnout	4.63	—

Henseler in 2009 proposed that CV‐RED should be assessed against three specific thresholds: 0.02, 0.15, and 0.35. Each threshold corresponds to a different level of quality: poor, moderate, and strong [35]. In this study, the CV‐Red values for all three variables fell within the moderate category, as indicated in Table 5.

**Table 5:** Cross‐validation redundancy.

	R Square	CV COM	CV RED
burnout	1.000	0.311	0.377
satisfaction	0.994	0.238	0.282
skill	0.999	0.239	0.349

### 3.5 Model Fit

The standardized root mean square residual (SRMR) is a measure of the discrepancy between the observed correlations and those predicted by the model. Generally, a SRMR value below 0.10 or 0.08 is indicative of a good fit. Henseler et al. (2014) recognized SRMR as an effective tool for assessing model fit in PLS‐SEM, helping to prevent model misinterpretations [37]. In this study, an SRMR value of 0.120 was observed, falling into the medium to high range, which suggests an adequate fit of the model.

### 3.6 Mediation Analysis

The investigation into the mediating role of job burnout in the relationship between job skills and job satisfaction revealed that the direct path remains significant, both with and without the inclusion of the mediating variable. However, subsequent analysis using variance accounted for (VAF) indicated a VAF of 0.023. According to Hair and et al. VAF values are interpreted as follows: a VAF greater than 80% suggests full mediation, a VAF between 20% and 80% suggests partial mediation, and a VAF less than 20% indicates no mediation [38]. Based on these criteria, the results suggest that job burnout does not play a mediating role in this context.

## 4 Discussion

In our model, we found that job skills are positively and significantly related to job burnout and job satisfaction. Also, job burnout in the relationship between job skills and job satisfaction revealed that the direct path remains significant, both with and without the inclusion of the mediating variable. However, according to Variance Accounted For, job burnout does not play a mediating role in this context.

### 4.1 The Role of Job Skills

Our findings show that there is a relationship between job skills and job satisfaction, which is consistent with the findings of Fernández‐Salinero et al. and Kucharska et al's research [7, 40]. Of course, this is contrary to the findings of some other studies. Some research suggests that people with higher skills tend to show lower levels of job satisfaction [39, 41]. Job skills have become a key factor in the recruitment process and human resource management [7]. Job skills are learned mental or motor behaviors required to facilitate the performance of complex tasks or actions. Therefore, skills and abilities are actual skills that are obtained from the application of potential abilities and talents as a result of experience and practice [29, 42].

As stated, the findings show a relationship between job skills and job satisfaction. Specifically, enhancing job skills substantially contributes to increased job satisfaction among employees. This positive impact, however, is partially offset by the phenomenon of job burnout. Job burnout dampens the otherwise positive influence of job skills on job satisfaction. This suggests that while proficiency in job skills can elevate an employee's sense of satisfaction and fulfillment, the benefits are somewhat mitigated if the individual is simultaneously experiencing high levels of burnout.

### 4.2 The Role of Job Burnout

While job burnout is present in the relationship between job skills and job satisfaction, it's not a mediator. This could be attributed to the direct and strong impact of job skills on job satisfaction, which appears to overshadow any mediation role of job burnout.

Based on some of the previously conducted research, people who experience burnout often report reduced job satisfaction, and cognitive functioning, with fatigue being a particularly notable issue [43, 44, 45, 46, 47, 48]. Job autonomy and job satisfaction were the significant determinants of burnout risks [49]. Because improving job satisfaction combined with increasing job autonomy may be effective in protecting from burnout [31].

Davali and Alipour's study shows that job skills play an essential role in enhancing job satisfaction. Also, this relationship is somewhat influenced by the presence of job burnout, which acts as a partial mediator. This suggests that while job skills directly contribute to increasing job satisfaction, the extent of this impact is somewhat moderated by the level of job burnout experienced by individuals [5].

According to Matthews and et al. job satisfaction is directly predicted by two dimensions of job burnout (personal accomplishment and emotional exhaustion). Personal accomplishment has a significant positive relationship with job satisfaction. Also, emotional exhaustion has a significant negative relationship with job satisfaction. Depersonalization had a significant positive relationship with job satisfaction but also produced a significant negative indirect relationship [50].

### 4.3 Potential Limitations and Suggestions for Future Research

Our study had several limitations due to the cross‐sectional design, which limits its ability to infer causality. Longitudinal studies are needed to establish a causal relationship between job skills, satisfaction, and burnout. Also, qualitative research, including interviews or focus groups, could provide deeper insights into the subjective experiences of employees regarding job skills, satisfaction, and burnout. Additionally, given the difficulty of work and different job conditions, the findings may not be generalizable to all industries or job roles. Further research is needed to determine if these findings hold across different sectors and levels of employment. Another major limitation is limited variables. The study focused on job skills, satisfaction, and burnout. Other variables, such as work environment, management style, and personal factors, were not considered and may also play a significant role. While this study did not find job burnout to be a mediator, future studies could explore other potential mediators, such as organizational support, work‐life balance, and employee engagement. Also, demographic variables were not included in the model in this study, and their effect on the dependent variable was not investigated. Future studies using specialized software, such as MPLUS, should also investigate the effect of demographic variables on the model.

## 5 Conclusion

This study contributes to understanding how job skills directly impact employee satisfaction and burnout. Job burnout's role as a mediator is not supported in this context. The results highlight the crucial role of job skills in boosting job satisfaction within health information management departments, which are increasingly important in the rapidly changing global healthcare landscape. Additionally, the study underscores the significance of effectively managing job burnout to maximize employee job satisfaction in the stressful environment of health information management. The findings enhance our understanding of job satisfaction, provide managers with a framework for identifying factors influencing job satisfaction, and suggest future research directions. In summary, our research contributes to the academic discussions on employee well‐being in the healthcare industry and offers practical recommendations for health information management departments worldwide. It presents specific approaches to enhance job satisfaction, focusing on skill development and mitigating job burnout.

## Author Contributions

**Mahdie Shojaei Baghini:** conceptualization, investigation, funding acquisition, writing–original draft, methodology, writing–review and editing, software, project administration. **Dorsa SepehryRad:** data curation, validation. **Fatemeh Miri:** data curation, software. **Fatemeh Asadi:** writing–original draft, formal analysis.

## Ethics Statement

The Research Ethics Committee of Kerman University of Medical Sciences issued the ethics code of the study: IR. KMU. REC.1402.026. The authors are accountable for all aspects of the work in ensuring that questions related to the accuracy or integrity of any part of the work are appropriately investigated and resolved.

## Conflicts of Interest

The authors declare no conflicts of interest.

## Transparency Statement

The lead author Mahdie Shojaei Baghini affirms that this manuscript is an honest, accurate, and transparent account of the study being reported; that no important aspects of the study have been omitted; and that any discrepancies from the study as planned (and, if relevant, registered) have been explained.

## Data Availability

No additional data are available. Mahdie Shojaei Baghini, the lead, and corresponding author, had full access to all the data in this study and takes complete responsibility for the integrity of the data and the accuracy of the data analysis. Data generated during the study are available on reasonable request through the corresponding author.
